# Medical education at the crossroads. Part I: undergraduate education and the erosion of professional identity

**DOI:** 10.1177/01410768251395407

**Published:** 2025-11-25

**Authors:** Louella Vaughan, Martin McKee

**Affiliations:** 1 Barts Health NHS Trust, London E1 1BB, United Kingdom; 2 Department of Internal Medicine, Amsterdam University Medical Centre, Amsterdam 1081 HV, The Netherlands; 3 London School of Hygiene & Tropical Medicine, London WC1H 9SH, United Kingdom

## Introduction

Undergraduate medical education (UGME) in the United Kingdom is undergoing a profound transformation. NHS England’s 10-Year Plan, *Fit for the Future*, published in 2025, outlines a strategic overhaul of healthcare delivery and training, driven by three core shifts: ‘hospital to community’, ‘analogue to digital’ and ‘sickness to prevention’.^1^ Central to this vision is the Neighbourhood Health Service model, which repositions care within communities and integrates health with broader social support systems. It was preceded by a 2023 document, the NHS Long Term Workforce Plan,^2^ and, although the 10-Year Plan rejects the earlier one, criticising its modelling as ‘fiction’, it remains in force for now. Together, they signal potentially significant changes to the education of current and future doctors.

In this pair of papers, we examine the implications of these plans for undergraduate and postgraduate medical education, while triangulating other recent policy announcements into the analysis. We have undertaken a ‘red teaming’ exercise,^3^ a method advocated and increasingly used by the government,^4^ in which we tested the resilience of proposals by examining plausible worst-case scenarios (Box 1).

**Box 1**

NHS strategic plans are typically high level and densely worded, with even brief statements capable of triggering wide-reaching changes across the healthcare system. These documents often carry unintended consequences, some of which may negatively affect medical education and service delivery. We undertook a process of ‘red teaming’, based on structured adversarial thinking that seeks to identify blind spots, challenge assumptions and simulate scenarios. Each author independently reviewed the Plan using close reading techniques^5^ to identify statements with potential implications for undergraduate and postgraduate medical education, distinguishing between explicit policy proposals and inferred consequences. We triangulated them with recent publications from NHS England, the General Medical Council (GMC)^1,2,6,7,8^ and other relevant bodies.^9^ Through iterative discussion, we developed plausible worst-case scenarios and explored their underlying causes.

In this first paper, we focus on UGME and proposals to shorten degrees, shift placements into the community and introduce digital platforms, with increased involvement from non-medical educators and commercial providers. In the companion piece, we consider the implications for postgraduate medical education.

Our starting point is that these plans are being taken forward against a backdrop of serious tensions at several levels. Medical school places are expanding, while universities are facing severe financial pressure, threatening their ability to deliver foundational sciences, such as biochemistry and physiology, that underpin medical knowledge.^10^ Clinical training is shifting away from hospitals towards overstretched community settings, where general practitioners are unwilling to take on additional responsibilities.^11^ The doctor’s role is being redefined as a generalist team leader, embedded in a digitally enabled system, at a time when scientific advances, for example in genomics, are vastly increasing the complexity of care.^12^

While this offers opportunities for innovation and responsiveness to changing circumstances, it also raises questions about the sustainability, equity and integrity of medical training, with a possible reshaping of professional identity, loss of academic rigour and damage to coherence of UGME.

## Erosion of the foundations of medical training

If implemented as intended, noting that some elements of the Workforce Plan have already been abandoned,^13^ these proposals would require a potentially fundamental reconfiguration of undergraduate medical education (UGME), with significant implications for the scientific depth and academic integrity of medical training. Central to this transformation is the shift towards shortened degrees and digital platforms.

Universities are expected to ‘review course length’ in light of technological advances and the transition to lifelong learning.^1^ This opens the door to accelerated programmes despite existing concerns about the pace and depth of learning.^14^

Changes to UGME must start from an agreement about what doctors need to know. Currently, this is set out in the UK’s Medical Licensing Assessment,^15^ but this can change. One view, expressed by the outgoing Medical Director of the GMC, is that mobile phones mean that doctors will no longer need a ‘huge repository of facts in [their] heads.’^16^ Instead, they are expected to be more reliant on care plans and digital support systems. Yet the combination of an ageing and multi-morbid population with accelerating scientific advances, illustrated by the statement in the 10-Year Plan that ‘Inexpensive gene sequencing will allow doctors to routinely diagnose and treat patients’,^1^ will greatly increase the complexity of care and require yet more knowledge and skill.

The 10-Year Plan advocates curricula reforms that will ‘promote acquisition and retention of generalist skills required for the Neighbourhood Health Service’. However, in an already crowded curriculum, any reduction in foundational subjects, such as anatomy, physiology and pathology, risks undermining students’ ability to engage in clinical reasoning and evidence-based practice.^12^ Simultaneously, proposals in other policy documents for interprofessional education during the pre-clinical period^17^ risk diluting the specificity of medical education, with evidence from New Zealand that it fails to meet the needs of medical students.^18^ While interdisciplinary learning has value, the pace and complexity of medical training demand a distinct trajectory, particularly in the early years.

Perhaps most disruptive proposals are for expansion of recorded lectures, asynchronous learning modules, and Massive Open Online Courses (MOOCs) as means to replace in-person instruction and lower costs.^19^ The 10-Year Plan contains an explicit invitation to alternative providers, including ‘world-class technology firms and academic institutions’ to provide content and says ‘Where existing providers are unable to move at the right pace, we may look to different institutions to ensure that the education market is responsive to employer needs’. This challenges the historical role of universities as custodians of disciplinary coherence and academic standards.^19^ The fragmentation of curricula into modular, externally sourced content risks eroding pedagogical integrity and weakening the intellectual formation of future doctors.

These changes also threaten the research capacity of the medical education system. Universities have long served as incubators for clinician-scientists, fostering inquiry and innovation. A curriculum focused on task-based competencies and immediate service readiness may discourage students from pursuing academic careers, further exacerbating the decline in clinical academics and weakening the United Kingdom’s position in global medical research.^20^

In these ways, the plans deprioritise the scientific and academic foundations of medicine. While digital platforms and modular learning offer scalability and flexibility, they must not come at the expense of depth, coherence and the intellectual rigour that underpin professional identity and public trust in the medical profession.

## Clinical training and professional identity

Current proposals seem likely to lead to a fundamental shift in the clinical component of medical training, moving away from hospital-based education towards community-centred placements within NHCs. This reorientation reflects broader system goals, emphasising prevention, chronic disease management and generalist care, but it also disrupts the traditional apprenticeship model that has long underpinned professional formation.^21^

Historically, clinical education has relied on immersive hospital experiences, where students learn through observation, supervised practice and mentorship from senior doctors.^22^ The new emphasis on decentralised care and generalist competencies risks narrowing the scope of clinical exposure, particularly in acute and specialist settings. Students may graduate with limited experience in managing complex, high-stakes scenarios, undermining their preparedness for hospital-based roles.

Simulation and digital tools are promoted as scalable alternatives to traditional bedside learning. While high-fidelity simulation can enhance procedural skills and standardise training,^23^ it cannot fully replicate the unpredictability and nuance of real-world clinical encounters. The increasing reliance on asynchronous learning and algorithmic decision support may further erode students’ ability to develop clinical judgement. This has implications for the development of mental ‘maps’ of disease, potentially hindering their ability to diagnose disease and manage complexity.^24^

A particularly concerning development is the reconceptualisation of students as service contributors who can ‘start delivering for patients as soon as [skills and competencies] are acquired rather than trainees waiting until the end of a formal training period’, suggesting that they would be signed off locally rather than formally assessed. This shift, coupled with changes to clinical placement tariffs, risks positioning students as workforce substitutes rather than learners, raising ethical and pedagogical concerns.

Supervisory structures are also under pressure. The expansion of medical school places is coinciding with a reduction in the number of clinical academics, with the GMC proposing ‘a significant increase in the supply of multidisciplinary educators’.^6^ While appropriate interdisciplinary education has value, the erosion of peer-to-peer mentorship and diminishing the role of doctors in training future colleagues threaten the continuity and integrity of professional identity.

Together, these changes redefine the role of the doctor. Rather than autonomous diagnosticians, future doctors are envisioned as generalist team leaders embedded in digitally enabled systems. Their authority is increasingly mediated by protocols and AI, challenging long-standing notions of clinical independence and responsibility.^25^

In these respects, current proposals risk fragmenting the educational experience, weakening professional identity and compromising the depth of clinical training. As the boundaries between education, service and technology blur, it is essential to safeguard the formative experiences that shape competent, compassionate and accountable doctors.

## Regulation and fragmentation

Another tension arises between the recent introduction of the national Medical Licensing Assessment, which the 10-Year Plan does not mention, and the move towards a more localised, employer-driven model of education. This reorientation could challenge long-standing principles of consistency, quality assurance and professional coherence in undergraduate medical training. Without clear benchmarks or summative evaluations, it becomes difficult to assess progress towards readiness for clinical practice or to compare outcomes across programmes. This fragmentation may erode public trust in the profession and challenge the ability of regulators, such as the GMC, to uphold consistent standards.

These reforms also carry implications for equity and mobility. If university curricula do diverge, it could reinforce informal hierarchies among medical schools, affecting perceptions of prestige and access to postgraduate opportunities.^26^ The Plan applies only to England, raising the possibility of divergence from medical schools in Scotland and Wales, and potentially creating barriers to interregional mobility.

International recognition is another concern. The UK medical degrees have long been highly valued globally, attracting international students and facilitating opportunities for practice abroad. If shortened degrees, localised standards and weakened quality assurance become the norm, the UK qualifications may no longer meet the criteria for registration in other jurisdictions, including the European Union.^27^ This could have economic consequences for universities and limit career options for graduates.

While proposed regulatory reforms aim to increase flexibility and responsiveness, they risk undermining the integrity and portability of medical education. A balance must be struck between local innovation and national coherence, ensuring that all medical graduates, regardless of where they train, are equipped to meet the demands of clinical practice and uphold the standards of the profession.

## Conclusion

The government’s proposals for the NHS in England present opportunities and risks for UGME. An emphasis on decentralisation, digital innovation and workforce flexibility reflects a desire to modernise training and align it more closely with evolving service needs. Community-based placements, simulation and modular learning offer potential efficiencies, while competency-based progression may support personalised development. However, they also raise serious concerns. The potential for erosion of foundational science and fragmentation of curricula threatens the coherence and rigour of medical education. The redefinition of the doctor’s role, from autonomous clinician to protocol-guided team leader, risks undermining professional identity and clinical judgement. The shift towards employer-driven standards and alternative providers may compromise equity, consistency and international recognition.

To navigate this transformation successfully, reform must be grounded in clarity and integrity. Flexibility should not come at the expense of depth, and innovation must be balanced with safeguards that preserve the intellectual, ethical and clinical foundations of the profession. Medical education must continue to produce doctors who are not only technically competent but also capable of critical thinking, compassionate care and leadership in complex environments. The future of the profession depends on getting this balance right.

## Declarations

**Ethical approval:** This article does not contain any studies with human or animal participants.

**Guarantor:** LV.

**Consent to participate:** Not applicable.

**Consent for publication:** Not applicable.
